# Mental health facility ownership and smoking cessation services: a facility-level analysis across the United States

**DOI:** 10.3389/fpubh.2025.1724032

**Published:** 2025-12-30

**Authors:** Abdullah A. Alharbi

**Affiliations:** Family and Community Medicine Department, Faculty of Medicine, Jazan University, Jazan, Saudi Arabia

**Keywords:** mental health facilities, smoking cessation, facility ownership, healthcare disparities and economics, service accessibility

## Abstract

**Background:**

In the U.S., tobacco use affects 19.8% of adults (49.2 million) as of 2022, with rates highest among those with mental health conditions. Mental health facilities offering cessation counseling present critical opportunities to support these vulnerable populations. This study aims to examine how ownership structure influences smoking cessation counseling provision in U.S. outpatient mental health facilities.

**Method:**

This cross-sectional study derived data on 9,645 outpatient (OPD) mental facilities from the 2019 N-MHSS—a census of all public and private mental health treatment facilities in the United States. We used multiple logistic regressions to examine how facility ownership would be associated with smoking cessation counseling provision, controlling for other facility characteristics. Models adjusted for state-level clustering for correlated random variances in service provisions across facilities in the same state.

**Result:**

Of the outpatient facilities surveyed, 41.72% of OPD provided smoking cessation counseling. Compared to public facilities, private for-profit facilities were 30% less likely to provide smoking cessation counseling [odds ratio (OR) = 0.70, 95% confidence interval (CI) = 0.59–0.82; *p* < 0.05]. Private non-profit facilities were 27% less likely to provide smoking cessation counseling (OR = 0.73, CI = 0.64–0.83; *p* < 0.05), while controlling for other confounders.

**Conclusion:**

Publicly-owned facilities demonstrate higher rates of smoking cessation counseling provision compared to both private non-profit and for-profit counterparts. This disparity in service availability raises concerns for tobacco control efforts, particularly as privatization in mental healthcare continues to expand. The observed pattern suggests potential misalignment between public health priorities and service delivery in private settings. Future policy initiatives should address these gaps and investigate underlying factors driving ownership-based differences in cessation services for patients with mental illness.

## Introduction

In the U.S., tobacco product use remains the leading cause of preventable disease and death. According to the CDC's analysis of 2019–2022 National Health Interview Survey data, 49.2 million adults (19.8%), or nearly 1 in 5, reported current tobacco product use in 2022, with cigarettes remaining the most commonly used tobacco product (1). Smoking causes 480,000 annual deaths, including 41,000 from secondhand smoke exposure. While smoking rates vary across socioeconomic status, education, and race/ethnicity, individuals with mental health conditions consistently show higher smoking rates compared to their counterparts without such conditions within each demographic group (2). For example, in 2016, while 25.3% of adults living below the poverty level smoked cigarettes, this rate nearly doubled to 48% among adults with mental health conditions who were also below the poverty level.

Smoking cessation remains a significant public health priority due to the documented economic costs and health consequences of tobacco use. Research indicates that a combination of medication, nicotine replacement therapy (NRT) and behavioral counseling therapy offers an effective approach to address nicotine addiction (3–7). Studies have attempted to determine what kind of and what intensity of interventions are helpful in smoking cessation. Smoking cessation counseling is appropriate behavioral and motivational counseling that is sustained over time (3–7). When smokers are only given nicotine replacement therapy or other pharmacological choices, the cessation rate is lower than when it is accompanied by counseling (5). A multi-year national study (from 2006 to 2012) in the USA demonstrated that the prevalence of Substance Abuse Treatment Facilities (SATFs) offering smoking cessation services increased over time, from 13 to 65%. The data also indicated that the ownership status of facilities (public vs. private) was one of the factors most strongly associated with the availability of these services (8).

Evidence demonstrates that smoking cessation is particularly crucial for individuals with severe mental conditions (SMD). Tobacco use is elevated more than five-fold in people with schizophrenia (9, 10) and is a leading preventable cause of death, contributing to cardiovascular disease, respiratory disorders, and cancers (11). People with SMD start smoking earlier and smoke more heavily than the general population (12), yet global tobacco reduction successes have not reached SMD populations (9, 13). Addressing substance use in SMD populations reduces all-cause mortality risk (14–16) and decreases psychiatric admissions, emergency presentations, and hospital stays (17). For healthcare facilities, smoking cessation services address a key modifiable risk factor for premature mortality while reducing healthcare utilization. Integrating smoking cessation aligns with WHO guidelines for “Management of physical health conditions in adults with severe mental disorders” (18), which aim to reduce health risk behaviors and mortality in SMD. By providing these services, facilities help break the cycle between mental and physical health conditions while addressing healthcare barriers.

The pharmacological aspect of the combination of pharmacotherapy with behavioral counseling employs many different types of drugs. These include nicotine replacement therapy (NRT) such as patches or gum (3–7), Varenicline (5) to block the receptors for tobacco, antidepressants such as Bupropion (4) and Nortriptyline (5), and nicotinic acetylcholine agonists such as Cytisine (5). Cognitive behavioral therapy is also used (3). Other interventions are to simply advise patients to quit smoking, written instructional informational materials given to smokers, and educational efforts aimed at schools and other organizations (5, 7). None of these alone have been as effective as those with smoking cessation counseling combined.

Despite critical needs to aid smoking cessation among mental health illness and the benefits of smoking cessation counseling, the national availability of smoking cessation counseling has not been comprehensively documented. Facilities with different ownership structures may operate with varying organizational priorities. For example, the clients/patients differ between public and private for-profit facilities as private for-profit facilities may be more likely to serve patients with private insurance or self-pay, whereas, both public and private non-profit see more patients on Medicaid. Public institutions may serve patients with more complex addiction profiles including those with multiple addictions, those who have been addicted longer, and those with fewer financial resources (19). Most private facilities are non-profit, but the for-profit private sector is growing and increasingly state and federal agencies are contracting with private for profit facilities (19). There may be some blurring of the lines between these three ownership types, for example many private non-profit facilities depend on private insurance and self-pay sources of revenue and may resemble private for-profit agencies in their planning and characteristics (20). Given these differences, it is important to examine facility ownership to see if ownership impacts whether smoking cessation counseling is offered.

Unequal distribution of smoking cessation counseling across mental health facilities may limit access for vulnerable populations. Indeed, one demographic which appears to have limited access to evidence based smoking cessation interventions are the mentally ill (21–23). Evidence suggests that access to smoking cessation counseling remains limited in the mental healthcare field for three primary reasons: one is that there are simply not enough mental healthcare workers, second is that there is no emphasis on smoking cessation in the mental healthcare field, and third mental healthcare workers are not trained in smoking cessation (21–23). In the U.S., access to resources for smoking cessation is variable and dependent on ownership type of the facility, whether it is publicly owned or privately owned and whether it is a for profit or non-profit facility. Mental healthcare workers, including psychiatrists do not screen patients regularly for smoking habits (23). Tobacco use among individuals with mental illness may receive insufficient clinical attention, yet the mental healthcare setting is the most logical place to address smoking cessation whether for returned veterans' PTSD, the severely mentally ill such as schizophrenics, or the patient with a substance abuse problem (21–23). These findings suggest a need for enhanced training of mental healthcare workers in assessing smoking and using resources to help the patient stop smoking (21). Facilities owned by different sectors might vary in the actions on intervening in such workforce training for smoking cessation services.

Cunningham (24), states that a survey of primary health practioners (PCPs) revealed barriers to access also include a shortage of available mental health services. These issues vary across facilities and communities resulting in a lack of parity which will affect access to some groups and locations. Low income is another barrier (25), who may disproportionately rely on services provided by public or private non-profit facilities. Yet, the unavailability of services on smoking cessation medications and counseling may hinder the access to comprehensive treatment support services (25). Knudsen et al. (26), found that organizational cultural, financial problems, and staffing issues presented barriers to the availability of comprehensive smoking cessation services related to facility ownership. Because the first step in providing smoking cessation services is the initial intake interview with the patient, they used the adoption of the Public Health Services five-point intake guideline as an indication of organizational cultural level of acceptance of the need for smoking cessation services.

This study examines the association between facility ownership (public, private non-profit, or private for-profit) and the provision of smoking cessation services in mental health outpatient departments nationwide in the USA. Despite the high prevalence of smoking among individuals with mental health conditions and substance abuse disorders, there exists a significant gap in research regarding how ownership status influences cessation counseling availability for this vulnerable population. Current literature lacks comprehensive analysis of this relationship, particularly in outpatient settings. By conducting the first systematic assessment of how ownership structures affect smoking cessation counseling across the United States, this research addresses a critical knowledge gap in tobacco control and mental health service delivery, potentially informing policy development to enhance service parity.

## Methodology

### Study design and data source

Data on nationwide mental health treatment facilities in the United States were obtained from the 2019 National Mental Health Services Survey (N-MHSS) conducted by the Substance Abuse and Mental Health Services Administration (SAMHSA). The N-MHSS is the only comprehensive national survey capturing both public and private mental health treatment facilities across all 50 states, the District of Columbia, and U.S. territories. This cross-sectional survey gathers information on location, characteristics (e.g., ownership), and service utilization across all 50 states, the District of Columbia, Puerto Rico, and other jurisdictions (27). The 2019 dataset was selected to capture pre-pandemic baseline conditions and avoid COVID-19-related confounding effects on service delivery. While more recent N-MHSS data are available (2024), the 2019 data represent the most recent comprehensive assessment of mental health facilities operating under standard conditions before pandemic-related disruptions. According to the 2019 survey, the final unit response rate among facilities eligible for the survey was 91 percent, representing 12,712 mental health treatment facilities out of 14,013 eligible facilities. The survey employed comprehensive quality assurance procedures to minimize potential non-response bias, including manual review for consistency and missing data, verification of questionable responses through automated reviews, and a web-based questionnaire programmed to prompt completion of missing responses. While this high response rate substantially reduces concerns about non-response bias, facilities that did not respond may differ systematically from respondents in ways that could affect our estimates. These data provide comprehensive information on various mental health services, including smoking cessation counseling. Our analysis specifically focused on outpatient mental health facilities (OPD) offering such services. After methodically excluding inpatient-only facilities, those providing only administrative services, and cases with missing data, our final analytical sample consisted of 9,645 outpatient mental health facilities (16).

### Measurements

The primary objective of this study was to examine the association between ownership status of outpatient mental health facilities and the provision of smoking cessation services (counseling) across mental health facilities (MHFs) in the United States. All variables used in our analysis were either binary or categorical. Our primary outcome was the provision of smoking cessation counseling, coded as a binary variable where 0 indicated that the facility did not provide counseling and 1 indicated the facility provided counseling. The main independent variable of interest was MHF ownership, categorized as follows: 0 for public agency or department, 1 for private for-profit organization, and 2 for private non-profit organization. For the logistic regression analysis, all categorical variables were converted to dummy variables. Ownership type was recoded into two dummy variables with public facilities serving as the reference category, allowing for comparisons of private for-profit and private non-profit facilities against public facilities. Covariates encompass various facility characteristics, including facility types, setting groups, primary focus areas, census divisions, and age groups served. Each multi-category covariate was similarly dummy-coded with one category designated as the reference group: psychiatric hospitals for facility type, Northeast for census division, mental health treatment for primary focus, and so forth. For binary variables, “No” (coded as 0) served as the reference category. This dummy variable approach enabled the estimation of odds ratios representing the likelihood of providing smoking cessation counseling for each category relative to its reference group. These factors are incorporated to provide a comprehensive view of mental health services. Additional covariates are also considered to ensure a thorough analysis of the facilities and their services (27). As this is the first study examining ownership-smoking cessation service associations using secondary N-MHSS data, covariates were selected based on data availability and prior literature suggesting their potential association with healthcare service provision in mental health settings.

### Statistical analysis

Our approach involved three steps. First, we performed descriptive analysis on MHF characteristics, including ownership type, geographical region, primary focus, age groups served (excluding those under twelve), and others. Next, Chi-square tests were conducted to assess associations between facility ownership status and smoking cessation counseling, as well as other covariate relationships. Lastly, multiple logistic regression was employed to identify which facility ownership types predicted smoking cessation counseling provision, while controlling for other covariates. Geographic variation was controlled using census divisions (Northeast, Midwest, South, West) rather than state fixed effects to maintain model parsimony while capturing regional differences in service delivery patterns. We calculated adjusted Odds ratios (aORs) and 95% confidence intervals (CIs). Data analysis was performed using Stata Statistics software, Version 16. All tests were two-sided, with statistical significance set at *p* < 0.05.

## Results

Table 1 shows smoking cessation counseling provision across 9,645 mental health facilities. Only 41.72% offered such services, with significant variation by ownership and facility type (*p* < 0.000). Private non-profits comprised 61% of facilities (38% providing cessation services), private-for-profits 18% (36% providing services), and public agencies 21% (57% providing services). Outpatient facilities represented 53% of the sample with low service provision rates (34%), while community mental health centers (27% of facilities) showed 40% provision rates. Veterans Administration centers showed the highest provision (91%), followed by inpatient psychiatric units (76%) and psychiatric hospitals (75%), though these represented only 12% of facilities.

**Table 1:** Characteristics of the study sample and distribution of MHF characteristics by provision of smoking cessation services in USA-2019.

**Characteristics**	**Total facilities 9,645 (100%)**	**No provision of smoking cessation services (counseling) 5,621 (58.28)**	**Provision of smoking cessation services (counseling) 4,024 (42)**	***p*-value**
**Faciltity charecterestics**				
**Facility ownership**				
Private-for-profit organization	1,769 (18)	1,137 (64)	632 (36)	< 0.001
Private non-profit organization	5,882 (61)	3,619 (62)	2,263 (38)	
Public agency or department	1,994 (21)	865 (43)	1,129 (57)	
**Facility types**				
Psychiatric hospitals (PH-facilities)	271 (3)	29 (25)	202 (75)	< 0.001
Separate inpatient psychiatric unit (SIPU-facilities)	403 (4)	96 (24)	307 (76)	
Residential treatment center (RTC-facilities)	149 (2)	101 (68)	48 (32)	
Veterans administration medical center (VAMC-facilities)	481 (5)	45 (9)	436 (91)	
Community mental health center (CMHC-facilities)	2,650 (27)	1,588 (60)	1,062 (40)	
Partial hospitalization/day treatment facility (PHP/DTF-facilities)	210 (2)	150 (71)	60 (29)	
Outpatient mental health facility (OMHF-facilities)	5,141 (53)	3,368 (66)	1,773 (34)	
Multi-setting mental health facility (MMHF-facilities)	340 (4)	204 (60)	136 (40)	
**Primary focus**				
Mental health treatment	5,751 (60)	3,864 (67)	1,887 (33)	< 0.001
Mix of mental health and substance abuse treatment	3,489 (36)	1,683 (48)	1,806 (52)	
General healthcare	405 (4)	74 (18)	331 (82)	
**Provision coverage by patient age**				
**Adolescents (13–17)**				
No	2,531 (26)	1,066 (42)	1,465 (58)	< 0.001
Yes	7,114 (74)	4,555 (64)	2,559 (36)	
**Young adults (18–25)**				
No	574 (6)	462 (80)	112 (20)	< 0.001
Yes	9,071 (94)	5,159 (57)	3,912 (43)	
**Adults (26–64)**				
No	1,119 (12)	945 (84)	174 (16)	< 0.001
Yes	8,526 (88)	4,676 (55)	3,850 (45)	
**Seniors (65 or older)**				
No	1,480 (15)	1,240 (84)	240 (16)	< 0.001
Yes	8,165 (85)	4,381 (54)	3,784 (46)	
**Payer acceptance**				
**Cash or self-payment**				
No	1,429 (15)	849 (59)	580 (41)	0.347
Yes	8,216 (85)	4,772 (58)	3,444 (42)	
No	1,714 (18)	1,113 (65)	601 (35)	< 0.001
Yes	7,931 (82)	4,508 (57)	3,423 (43)	
**Medicare**				
No	2,810 (29)	1,999 (71)	811 (29)	< 0.001
Yes	6,835 (71)	3,622 (53)	3,213 (47)	
**Medicaid**				
No	1,078 (11)	533 (49)	545 (51)	< 0.001
Yes	8,567 (89)	5,088 (59)	3,479 (41)	
**Settings**				
**Setting-inpatient**				
No	8,840 (92)	5,435 (61)	3,405 (39)	< 0.001
Yes	805 (8)	186 (23)	619 (77)	
**Setting-24-h residential**				
No	9,136 (95)	5,387 (59)	3,749 (41)	< 0.001
Yes	509 (5)	234 (46)	275 (54)	
**Setting-partial hospitalization/day treatment**				
No	8,232 (85)	4,916 (60)	3,316 (40)	< 0.001
Yes	1,413 (15)	705 (50)	708 (50)	
**Census division**				
West	2,305 (23.90)	1,496 (64.90)	809 (35.10)	< 0.001
Mid-west	2,494 (25.86)	1,473 (59.06)	1,021 (40.94)	
South	2,841 (29.46)	1,710 (60.19)	1,131 (39.81)	
North	2,005 (20.79)	942 (46.98)	1,063 (53.02)	

Further analysis of service provision patterns revealed significant variations in smoking cessation service provision across patient age groups and payment types (*p* < 0.000). Facilities serving young adults (94%) and adults (88%) were most common, while seniors had the highest cessation services provision rate (46%). Medicaid-serving facilities were most prevalent (89%), with Medicare-serving facilities showing the highest cessation service provision rate (47%).

Additionally, treatment setting and geographic location significantly influenced cessation service availability (*p* < 0.000). Inpatient facilities (8% of total) had the highest cessation services rate (77%), with partial hospitalization/day treatment settings showing balanced provision (50%). The North Census Division had the highest provision rate (53.02%), while the West had the lowest (35.10%).

Table 2 shows distribution of MHF characteristics by Facility Ownership in USA-−2019, with significant variation by ownership (*p* < 0.001). Public facilities had the highest smoking cessation counseling provision (56.6%; vs. 38.5% non-profit; 35.7% for-profit) and the greatest VAMC representation (24%; 0% in both private groups), with CMHCs at 29% (highest non-profit 32%; lowest for-profit 10%). For care settings, for-profits were most outpatient-oriented (OMHF 67%; public 39%; non-profit 54%) and had the highest partial hospitalization/day treatment (24%; public 11%; non-profit 13%), while inpatient availability was highest in for-profits (12%), lowest in non-profits (7%), with public in-between (10%); 24-h residential was highest in public (7%) vs. 5% in both private groups. Payer acceptance patterns showed non-profits highest for Medicaid (95%) and Medicare (75%); for-profits highest for cash (92%) and private insurance (87%); and publics lowest for cash (70%) and private insurance (72%) but moderate for Medicare (70%) and high for Medicaid (76%).

**Table 2:** Characteristics of the study sample and distribution of MHF characteristics by facility ownership in USA-2019.

**Characteristics**	**Total 9,645 (100%)**	**Private-for-profit 1,769 (18.35%)**	**Private non-profit 5,882 (60.98%)**	**Public agency/department 1,994 (20.67%)**	***p*-value**
**Provision of smoking cessation services**					
No	5,621 (58.28)	1,137 (64.27)	3,619 (61.53)	865 (43.38)	
Yes	4,024 (41.72)	632 (35.73)	2,263 (38.47)	1,129 (56.62)	
**Facility types**					
Psychiatric hospitals (PH)	271 (2.81)	158 (8.93)	73 (1.24)	40 (2.01)	< 0.001
Separate inpatient psychiatric unit (SIPU)	403 (4.18)	54 (3.05)	292 (4.96)	57 (2.86)	
Residential treatment center (RTC)	149 (1.54)	36 (2.04)	100 (1.70)	13 (0.65)	
Veterans administration medical center (VAMC)	481 (4.99)	0 (0.00)	0 (0.00)	481 (24.12)	
Community mental health center (CMHC)	2,650 (27.48)	169 (9.55)	1,908 (32.44)	573 (28.74)	
Partial hospitalization/day treatment (PHP/DTF)	210 (2.18)	85 (4.80)	101 (1.72)	24 (1.20)	
Outpatient mental health facility (OMHF)	5,141 (53.30)	1,182 (66.82)	3,178 (54.03)	781 (39.17)	
Multi-setting mental health facility (MMHF)	340 (3.53)	85 (4.80)	230 (3.91)	25 (1.25)	
**Primary focus**					
Mental health treatment	5,751 (59.63)	1,006 (56.87)	3,678 (62.53)	1,067 (53.51)	< 0.001
Mix of MH and substance use treatment	3,489 (36.17)	759 (42.91)	2,058 (34.99)	672 (33.70)	
General healthcare	405 (4.20)	4 (0.23)	146 (2.48)	255 (12.79)	
**Provision coverage by patient age**					
**Adolescents (13–17)**					
No	2,531 (26.24)	342 (19.33)	1,243 (21.13)	946 (47.44)	< 0.001
Yes	7,114 (73.76)	1,427 (80.67)	4,639 (78.87)	1,048 (52.56)	
**Young adults (18–25)**					
No	574 (5.95)	79 (4.47)	399 (6.78)	96 (4.81)	0.0001
Yes	9,071 (94.05)	1,690 (95.53)	5,483 (93.22)	1,898 (95.19)	
**Adults (26–64)**					
No	1,119 (11.60)	93 (5.26)	848 (14.42)	178 (8.93)	< 0.001
Yes	8,526 (88.40)	1,676 (94.74)	5,034 (85.58)	1,816 (91.07)	
**Senior**					
No	1,480 (15.34)	217 (12.27)	1,062 (18.06)	201 (10.08)	< 0.001
Yes	8,165 (84.66)	1,552 (87.73)	4,820 (81.94)	1,793 (89.92)	
**Payer acceptance**					
**Cash/self-payment**					
No	1,429 (14.82)	139 (7.86)	684 (11.63)	606 (30.39)	< 0.001
Yes	8,216 (85.18)	1,630 (92.14)	5,198 (88.37)	1,388 (69.61)	
**Private health insurance**					
No	1,714 (17.77)	238 (13.45)	918 (15.61)	558 (27.98)	< 0.001
Yes	7,931 (82.23)	1,531 (86.55)	4,964 (84.39)	1,436 (72.02)	
No	2,810 (29.13)	728 (41.15)	1,488 (25.30)	594 (29.79)	< 0.001
Yes	6,835 (70.87)	1,041 (58.85)	4,394 (74.70)	1,400 (70.21)	
**Medicaid**					
No	1,078 (11.18)	300 (16.96)	299 (5.08)	479 (24.02)	< 0.001
Yes	8,567 (88.82)	1,469 (83.04)	5,583 (94.92)	1,515 (75.98)	
**Settings**					
**Inpatient (hospital, 24-h)**					
No	8,840 (91.65)	1,556 (87.96)	5,496 (93.44)	1,788 (89.67)	< 0.001
Yes	805 (8.35)	213 (12.04)	386 (6.56)	206 (10.33)	
**24-h residential**					
No	9,136 (94.72)	1,683 (95.14)	5,589 (95.02)	1,864 (93.48)	0.020
Yes	509 (5.28)	86 (4.86)	293 (4.98)	130 (6.52)	
**Partial hospitalization/day treatment**					
No	8,232 (85.35)	1,344 (75.98)	5,104 (86.77)	1,784 (89.47)	< 0.001
Yes	1,413 (14.65)	425 (24.02)	778 (13.23)	210 (10.53)	
**Census division**					
West	2,305 (23.90)	473 (26.74)	1,348 (22.92)	484 (24.27)	< 0.001
Mid-west	2,494 (25.86)	397 (22.44)	1,668 (28.36)	429 (21.51)	
South	2,841 (29.46)	680 (38.44)	1,430 (24.31)	731 (36.66)	
North	2,005 (20.79)	219 (12.38)	1,436 (24.41)	350 (17.55)	

Table 3 presents our main findings examining the association between facility ownership (public, private non-profit, or private for-profit) and the provision of smoking cessation services in mental health outpatient departments nationwide in the USA. Focusing specifically on this ownership-provision relationship while controlling for all other facility characteristics, we found that compared to public agencies, the provision of cessation counseling was 30% less likely in for-profit private (PFP) facilities (aOR = 0.70, CI = 0.59–0.82, *p* ≤ 0.001), and 27% less likely in non-profit private (NPFP) facilities (aOR = 0.73, CI = 0.64–0.83, *p* ≤ 0.001).

**Table 3:** Multiple logistic regression analysis of mental health facility factors associated with provision of smoking cessation services in USA-2019.

**Characteristics**	**OR**	***p*-value**	**95% Conf interval**	
			**Lower**	**Upper**
**Faciltity charecterestics**				
**Facilities ownership**				
Base public agency or department	1			
Private for-profit	0.70	0.00^***^	0.59	0.82
Private non-profit	0.73	0.00^***^	0.64	0.83
**Facility types**				
Base psychiatric hospitals (PH-facilities)	1			
SIPU-facilities	0.90	0.569	0.61	1.31
RTC-facilities	0.48	0.052	0.23	1.01
VAMC-facilities	2.89	0.001^**^	1.53	5.41
CMHC-facilities	0.349	0.00^***^	0.20	0.61
PHP/DTF-facilities	0.27	0.00^***^	0.14	0.50
OMHF-facilities	0.30	0.00^***^	0.17	0.51
MMHF-facilities	0.35	0.001^**^	0.19	0.64
**Primary focus**				
Base Mental health treatment	1			
Mix	2.19	0.00^***^	1.98	2.42
General healthcare	4.49	0.00^***^	3.34	6.02
**Provision coverage by patient age**				
Adolescents (13-17)	0.67	0.00^***^	0.59	0.76
Young adults (18-25)	1.51	0.005^**^	1.12	2.01
Adults (26-64)	1.22	0.24	0.88	1.68
Seniors (65 or older)	1.68	0.00^***^	1.29	2.18
**Payer acceptance**				
Cash or self-payment	1.14	0.149	0.95	1.37
Private health insurance	1.10	0.23	0.94	1.28
Medicare	2.03	0.00^***^	1.78	2.32
Medicaid	1.05	0.65	0.86	1.27
**Settings**				
Setting-inpatient	1.66	0.03^*^	1.03	2.68
Setting-24-h residential	1.31	0.09	0.953	1.79
Setting-partial hospitalization/day treatment	0.99	0.91	0.85	1.15
**Census division**				
Base west	1			
Mid-west	1.001	0.99	0.86	1.14
South	0.94	0.33	0.82	1.07
North	2.11	0.00^***^	1.83	2.43

Variables marked with “base” use designated reference groups (OR = 1), while other variables represent binary yes/no comparisons for each characteristic.

^*^Significant difference at *p* ≤ 0.05.

^**^Significant difference at *p* ≤ 0.01.

^***^Significant difference at *p* ≤ 0.001.

## Discussion

This is a cross sectional study to assess the availability of smoking cessation counseling across 9,645 U.S. outpatient mental health facilities by ownership type. Our findings reveal significant disparities, with public facilities offering more cessation services than private for-profit and non-profit facilities. This gap is concerning given that mental health patients consume 44% of cigarettes in the US and face higher smoking-related mortality (28, 29). A study by Patten et al. (30), found that patients who had been in inpatient psychiatric hospitals where a smoking ban was enforced returned to their previous smoking behavior after discharge and the study suggested that more intensive treatments should be offered. This study offers evidence on the disproportion of smoking cessation counseling services across outpatient mental healthcare settings by ownership.

Our findings show substantial ownership-based disparities in smoking cessation counseling across U.S. mental health facilities. Of the total sample, 41.7% provided smoking cessation counseling services. The ownership distribution was 61% private non-profit, 21% public, and 18% private for-profit. While factors behind these differences aren't entirely clear, our findings align with previous studies showing public substance abuse facilities offer more comprehensive smoking cessation treatments than private for-profit counterparts (31).

Morever, our adjusted multiple logistic regression analysis revealed that for-profit private (PFP) facilities were 30% less likely, and non-profit private (NPFP) facilities were 27% less likely to provide cessation counseling compared to public facilities. These results align with a previous study in the USA, which found that public facilities had the highest prevalence of providing smoking cessation services. Although that study focused on general services, while ours specifically examined counseling, our findings remain strongly applicable and highlight the innovative aspect of our research (8).

Existing research on tobacco control methods in relation to facility characteristics is limited. The majority of studies have focused on assessing smoking bans and their effectiveness, primarily in inpatient hospital and psychiatric settings (32–34). Hospital ownership is a key determinant in understanding variations in hospital performance under prospective payment systems. It is well known that type of ownership and financing system are important factors in explaining how hospitals operate, which services they offer and to whom these services are available (8).

Our findings show public facilities provide smoking cessation services more frequently than private facilities, which may reflect differences in organizational priorities. Public mental health facilities may prioritize comprehensive, population-based care including substance use prevention, while private for-profit facilities focus on specialized services with higher profit margins. Smoking cessation programs require substantial resources (counseling, staff training, long-term follow-up) with limited financial returns, potentially making them less appealing to profit-driven facilities (35, 36). This aligns with broader patterns where private hospitals select less complex cases and limit resource-intensive services to maintain profitability (35, 37). Research shows that private for-profit hospitals are more likely to offer expensive, profitable services like surgical treatments compared to non-profit hospitals, which in turn are more inclined to do so than public hospitals (36, 38). This pattern suggests that public facilities, less constrained by profit motives, may prioritize comprehensive healthcare including preventive and counseling services. In contrast, private facilities tend to focus on specialized, more lucrative services like surgery. This disparity underscores the need for policies to ensure equitable access to all healthcare services, including preventive and counseling, across various facility types. Additionally, the higher prevalence of smoking cessation services in public facilities compared to private ones might be attributed to financial support from federal and state bodies, including State Mental Health Agencies and the US Department of Veterans Affairs (38).

Our review also discovered some apparent barriers to the provision of comprehensive smoking cessation counseling. Organizational management characteristics and staff beliefs about smoking cessation may be associated with service implementation, though our data cannot directly establish these relationships. Further research is needed to examine how management practices and organizational factors contribute to ownership-based disparities in service provision. A thorough knowledge of The Public Health Service (PHS) guidelines for smoking cessation strategy is lacking in most facilities but when it is present it is an indicator of the commitment of the facility to providing the best smoking cessation programs. Knudsen et al. (39) showed that privately owned facilities tended to implement the PHS intake guidelines at a higher rate than the public facilities (26). However, intake procedures are only part of the picture, the rate of smoking cessation strategies that adhere to the PHS guidelines for smoking cessation which is apart from the intake process is crucial for implementation.

Other factors such as staffing and financial considerations revealed that staffing is lower in private for-profit facilities and the veterans administration medical centers than in state and local public facilities or private non-profit facilities (19). Inadequate staffing may limit a facility's capacity to provide smoking cessation procedures. Private for-profit facilities may have concerns about reimbursement for staff time to implement smoking cessation procedures (26). One indication that there may be inequities in this system is to compare the patient demographics between public, private for profit, and private non-profit. Private for profits tend to treat patients who are employed and insured, live in the suburbs, have less severe addictive behaviors such as duration and numbers of substances abused, and to have a higher income bracket. Public facilities are more likely to see the most severe cases, patients with no insurance, veterans, and Medicaid patients (19). Richter et al. (40), found that facilities that employed specific written guidelines were more likely to implement smoking cessation procedures. Private for-profit facilities are less likely to provide smoking cessation treatment than public or private non-profit facilities. In light of the fact that private for-profit facilities are the fastest growing ownership type now, a continuation of such maldistribution of smoking cessation services might worsen the prevalence of smoking-related morbidity and mortality among mentally ill patients. Richter's study recommended that there be regulation requiring smoking cessation treatment for facilities treating drug addiction (40).

These findings have important policy implications. Regulations should require all outpatient mental health facilities to provide a minimum standard of smoking cessation counseling, ensuring consistent access across facility types. Incentive programs for private facilities should be introduced to encourage cessation counseling provision, aligning their practices with public agencies. These could include financial rewards or recognition for meeting standards. Partnerships between public and private facilities should be fostered to share best practices and resources. This collaboration could involve knowledge-sharing platforms, joint training sessions, and shared resource development, ultimately improving cessation support quality and availability for all patients.

Further research is recommended to conduct studies incorporating patient-level data, aiming to understand how demographic and clinical factors interact with geographic location to influence smoking cessation counseling rates. Additionally, it is suggested to investigate smoking cessation counseling rates in detail based on facility type and primary focus. Finally, further investigation is needed to examine policies and intervention programs that enhance smoking cessation counseling in facilities. These research directions would provide a more comprehensive understanding of the factors affecting smoking cessation counseling and help identify effective strategies to improve its implementation across various healthcare settings. Future studies should incorporate patient-reported outcomes and experiences to examine whether facility ownership differences translate into meaningful variations in patient satisfaction, treatment engagement, and cessation outcomes. Future studies should examine whether ownership effects on smoking cessation services vary by region, facility type, or payer mix through focused interaction analyses.

### Strengths and limitations

This study is among the first to examine smoking cessation services in mental health facilities nationally, considering ownership and potential predictors. It underscores the importance of a national monitoring system for mental healthcare access and improvement planning. The study's validity is high due to the N-MHSS dataset's size, valuable variables, frequent use in research, and long-term reliability. However, the N-MHSS is voluntary, potentially introducing non-response bias, and data from participating facilities are not adjusted for non-response. *Despite the high response rate of 91%, non-responding facilities may differ systematically in ownership structures or service provision patterns that could affect our estimates of smoking cessation counseling availability*. While the cross-sectional design precludes causal inferences, it addresses a gap in existing literature on mental health facilities and smoking cessation services. What we cannot determine from our data is that since the survey is of facilities, we have no way of knowing how many patients visit those facilities nor how many take advantage of smoking cessation counseling services. We also have no information about whether patients who have Serious Mental Illness (SMI), or substance abuse or addiction conditions utilize counseling services for smoking cessation. Using census divisions rather than state fixed effects may not fully capture state-level policy variations that could influence ownership patterns and service provision. Additionally, this study focused on main effects and did not examine potential interactions between ownership and other facility characteristics. Thus, future research should investigate whether ownership effects on smoking cessation services vary by geographic region, facility type, or patient populations served, as such interaction analyses could reveal important nuances in service delivery patterns. Finally, our facility-level analysis cannot capture patient perspectives, preferences, or satisfaction with smoking cessation services, which are essential for understanding the real-world impact of ownership-related differences in service provision.

## Conclusions

We analyzed data from the 2019 National Mental Health Services Survey to study the provision of smoking cessation counseling in outpatient mental health facilities. We found that public institutions provide higher rates of smoking cessation counseling than private institutions, especially private for-profit ones. However, the overall rate of smoking cessation counseling in outpatient mental health facilities is low especially in light of the higher rates of smoking among mental health patients and the need for smoking cessation. In addition to public and private ownership, other facility characteristics such as facility type and accepted payment also play a role on the probability of offering smoking cessation services. Our results should lead policy makers to consider ownership of facilities and the changing nature of public facilities when deciding to contract out mental health services to the private for profit sector. Policy makers should also consider whether there is a need for some requirements for mental health facilities to provide smoking cessation counseling so that everyone who needs it has access.

## Data Availability

Publicly available datasets were analyzed in this study. This data can be found at: https://www.samhsa.gov/data/data-we-collect/n-mhss-national-mental-health-services-survey.
